# Estrogens Regulate Placental Angiogenesis in Horses

**DOI:** 10.3390/ijms222212116

**Published:** 2021-11-09

**Authors:** Shingo Haneda, Pouya Dini, Alejandro Esteller-Vico, Kirsten E. Scoggin, Edward L. Squires, Mats H. Troedsson, Peter Daels, Yasuo Nambo, Barry A. Ball

**Affiliations:** 1 Department of Veterinary Medicine, Obihiro University of Agriculture and Veterinary Medicine, Obihiro 080-8555, Japan; haneda@obihiro.ac.jp (S.H.); ynambo@obihiro.ac.jp (Y.N.); 2 Gluck Equine Research Center, Department of Veterinary Science, University of Kentucky, Lexington, KY 40546, USA; pdini@ucdavis.edu (P.D.); a.esteller-vico@utk.edu (A.E.-V.); kirsten.scoggin@uky.edu (K.E.S.); edward.squires@uky.edu (E.L.S.); m.troedsson@uky.edu (M.H.T.); 3 Faculty of Veterinary Medicine, Ghent University, Salisburylaan 133, 9820 Merelbeke, Belgium; peter.daels@ugent.be; 4 Department of Population Health and Reproduction, School of Veterinary Medicine, University of California, Davis, Davis, CA 95616, USA; 5 Department of Biomedical and Diagnostic Sciences, College of Veterinary Medicine, University of Tennessee, Knoxville, TN 37996, USA

**Keywords:** chorioallantois, placenta, letrozole, angiogenesis, placental development, estrogen, 17β-estradiol, pregnancy, equine

## Abstract

A sufficient vascular network within the feto-maternal interface is necessary for placental function. Several pregnancy abnormalities have been associated with abnormal vascular formations in the placenta. We hypothesized that growth and expansion of the placental vascular network in the equine (*Equus caballus*) placenta is regulated by estrogens (estrogen family hormones), a hormone with a high circulating concentration during equine gestation. Administration of letrozole, a potent and specific inhibitor of aromatase, during the first trimester (D30 to D118), decreased circulatory estrone sulfate concentrations, increased circulatory testosterone and androstenedione concentrations, and tended to reduce the weight of the fetus (*p* < 0.1). Moreover, the gene expression of *CYP17A1* was increased, and the expression of androgen receptor was decreased in the D120 chorioallantois (CA) of letrozole-treated mares in comparison to that of the control mares. We also found that at D120, the number of vessels tended to decrease in the CAs with letrozole treatment (*p* = 0.07). In addition, expression of a subset of angiogenic genes, such as *ANGPT1*, *VEGF*, and *NOS2*, were altered in the CAs of letrozole-treated mares. We further demonstrated that 17β-estradiol increases the expression of *ANGPT1* and *VEGF* and increases the angiogenic activity of equine endothelial cells in vitro. Our results from the estrogen-suppressed group demonstrated an impaired placental vascular network, suggesting an estrogen-dependent vasculogenesis in the equine CA during the first trimester.

## 1. Introduction

Angiogenesis is the process of generating new blood vessels from a pre-existing vascular network to support tissue vascularization [1,2,3]. In general, the primary stimulus for angiogenesis is a lack of oxygen or nutrients in the surrounding tissue, which causes the non-vascular cells to produce angiogenic factors [4]. In adults, the process of angiogenesis is mainly limited to conditions such as restoring a tissue’s blood supply in response to injury or promoting wound healing, or it can be seen in developing tumors [5,6,7,8]. In addition, angiogenesis is one of the most important processes during the menstrual cycle in women, and angiogenesis is critical during pregnancy and placental development in eutherian mammals [2,5,9].

An adequate vascular network within the feto-maternal interface is a prerequisite for optimal placental function, normal fetal growth and development, and fetal and maternal lifelong well-being [9,10]. Moreover, several conditions, such as increased risk of miscarriage, preterm delivery, preeclampsia, hydrops, and intrauterine growth restriction (IUGR) have been associated with abnormal vascular formation in the placenta [11,12,13,14]. It is also known that the placental vascular network evolves throughout gestation to accommodate increasing fetal demands [15]. Accordingly, there is a positive association between the size of the fetus and the vascular network of the placenta [14]. The growth and expansion of the placental vascular network is caused by the branching of existing vessels, which includes the formation of new vessels through the sprouting of pre-existing vessels and the resulting rise in the number of capillaries [16].

It has been suggested that angiogenic activities in reproductive tissues are highly regulated by steroid hormones [16,17]. Powazniak et al. demonstrated that 17β-estradiol enhanced human endothelial cell proliferation, suggesting a promoting influence of 17β-estradiol on angiogenesis [18]. Moreover, it has been shown that 17β-estradiol increases the migration of endothelial cells through binding to the estrogen receptors, leading to the formation of new vessels [2,19,20]. It has also been demonstrated that 17β-estradiol suppresses expression of the matrix metalloproteinase inhibitor in the mouse uterus, facilitating the cleavage of the extracellular matrix and the formation of new vessels [21]. These effects of estrogens on angiogenesis are believed to be associated with the vascular endothelial growth factor (*VEGF*) expression [22,23]. This has been confirmed in human breast tissue, in which 17β-estradiol increases the extracellular levels of *VEGF* [23]. Furthermore, through the modulation of *VEGF* via a paracrine mechanism, 17β-estradiol increased vascular endothelial growth factor receptor-2 (*VEGFR2* (*Flk-1/KDR*)) expression in microvascular endothelial cells [22,24]. *VEGF* also stimulates the expression of endothelial nitric oxide synthase (*eNOS*) production, thus inducing vasodilatation and promoting endothelial cell proliferation [25]. It has also been reported that 17β-estradiol regulates expression of angiopoietin-1 (*ANGPT-1*), playing important roles in vascular development and function [26,27]. Even though many studies have described the effects of estrogens on angiogenesis, their effect on the equine placenta remains to be investigated.

Equine gestation is characterized by high circulating concentrations of estrogens (estrane or estrogen family hormones). The physiological roles of estrogens during gestation are largely unknown, although some studies suggest a relationship between low circulating estrogen concentrations and pregnancy loss in the mare [28,29,30]. Increases in circulating estrogens are derived from multiple sources during pregnancy. The equine conceptus begins estrogen secretion as early as D10 to 12 of pregnancy [31]. Beginning around D40 of gestation, increasing concentrations of equine chorionic gonadotropin (eCG) stimulate the formation of accessory corpora lutea as well as an increase in peripheral estrogen concentration in the mare, which appears to originate primarily from the corpora lutea at this time [32,33,34,35]. Later, at D60 to 90 of pregnancy, peripheral estrogens in the mare appear to be derived from the feto-placental unit, reaching a peak concentration at approximately D210 of gestation [36,37]. Production of estrogens beyond D60 during equine pregnancy appears to involve participation of both the fetal gonads and the placenta, termed the feto-placental unit. Androgens, primarily dehydroepiandrosterone (DHEA) from the fetal gonads, are finally aromatized in the placenta (chorioallantois) to form a variety of estrogens, including estradiol-17β, estrone, and the B-ring unsaturated estrogens equilin and equilenin [36,37,38]. However, the physiological role of elevated estrogens during pregnancy in mares is unknown. In primates, elevations in maternal estrogens are also noted during pregnancy, although the importance of estrogens in pregnancy maintenance is controversial [24]. The suppression of estrogens during early gestation in primates leads to a high rate of fetal loss accompanied by changes in placental vascularization during early pregnancy [39,40]. Here, we hypothesize that estrogen plays a pivotal role in the formation of blood vessels in the equine placenta.

The availability of potent and specific inhibitors of aromatase, such as letrozole, allow inhibition of the enzyme responsible for conversion of androgens to estrogens in the placenta. The use of letrozole and related aromatase inhibitors has facilitated investigating the importance of estrogens during pregnancy in animal models. As in reports regarding primates, inhibition of aromatase during late pregnancy in mares was found to result in a marked suppression of estrogens [39,41]. Moreover, our laboratory previously demonstrated that mares treated with letrozole had smaller foals at birth, which may reflect alterations in nutrient supply to the fetus due to the abnormal placenta [41]. In the aforementioned study, even though the number of vessels in the placenta was not evaluated, we believe that the restriction in the growth of the fetuses was associated with abnormal angiogenesis in these placentas. Similar associations between abnormal placental angiogenesis and fetal size have been reported in infants affected with IUGR [10,13,42]. To date, no reported studies have examined the effect of estrogen suppression on angiogenesis and fetal size during early pregnancy in mares.

In the current study, we hypothesized that estrogen suppression during early pregnancy in mares would affect angiogenesis in the placenta and, consequently, the size of the fetus. Therefore, we aimed to evaluate the role of estrogens in placental angiogenesis by using the aromatase inhibitor letrozole to suppress maternal estrogen synthesis between D30 and D120 of pregnancy, which is the critical period of placental development. We further aimed to evaluate the effect of 17β-estradiol on the angiogenic activity of equine endothelial cells in an in vitro assay.

## 2. Results

### 2.1. Letrozole-Treated Fetuses Tended to Weigh Less than Control Fetuses

**Figure 1:**
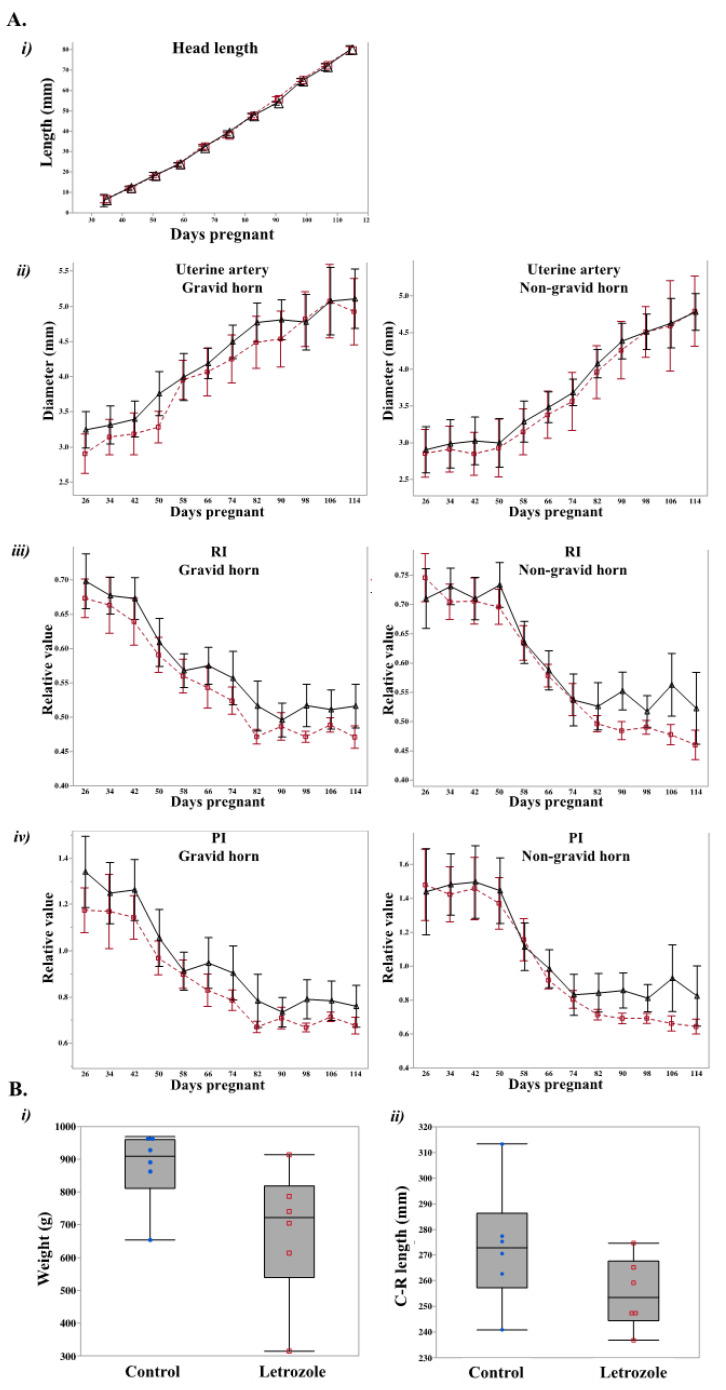
(**A**) Sequential change in parameters by ultrasonic examination. (***i***) Fetal head lengths of letrozole (black triangle) and control (red square) groups. (***ii***) Uterine artery diameter, (***iii***) resistance index (RI) and (***iv***) pulsatility index (PI) for both pregnant and non-pregnant uterine horns (gravid and non-gravid). No statistically significant differences or trends were observed among the groups (*p* > 0.5) (**B**) Fetal parameters on D120 are represented with box plots. Weights (***i***) and C–R lengths (***ii***) were compared between the letrozole and the control group. The letrozole-treated fetuses tended to weigh less than the control fetuses (*p* < 0.1) Red boxes (letrozole) and blue circles (control) represent individual observations.

Pregnant mares (*n* = 11, D26 (D0: day of ovulation)) were randomly assigned to either the letrozole-treated (*n* = 5) or control (*n* = 6) group. Letrozole-treated mares received 500 mg letrozole orally every four days, as previously established [26], and control mares received an equivalent mass of glucose orally every four days from D30 to D118 of gestation. Pregnancy was maintained in all control and treated mares up to the end of the study (D120). The fetal head lengths were measured from D42 until the end of the study. Although head length increased (*p* < 0.05) with time, no effect of treatment was detected (Figure 1A). The diameter of uterine arteries also increased (*p* < 0.05) with time, irrespective of the treatment (Figure 1A). Furthermore, treatment had no effect on the uterine artery resistance index (RI) or the pulsatility index (PI). However, both indexes decreased with gestational age (*p* < 0.05; Figure 1A).

At D120 of gestation, the fetus and placenta were collected transcervically from control and treated mares, and fetal weight and crown–rump (C–R) length were measured. The letrozole-treated fetuses tended to weigh less than the control fetuses (*p* < 0.1; Figure 1B). As for the results obtained by ultrasound scanning of the fetus, there were no differences in C–R length between treatments (Figure 1B).

### 2.2. Letrozole Treatment Altered Level of Peripheral Steroid Hormones and Steroidogenic Gene Expression in Chorioallantois

**Figure 2:**
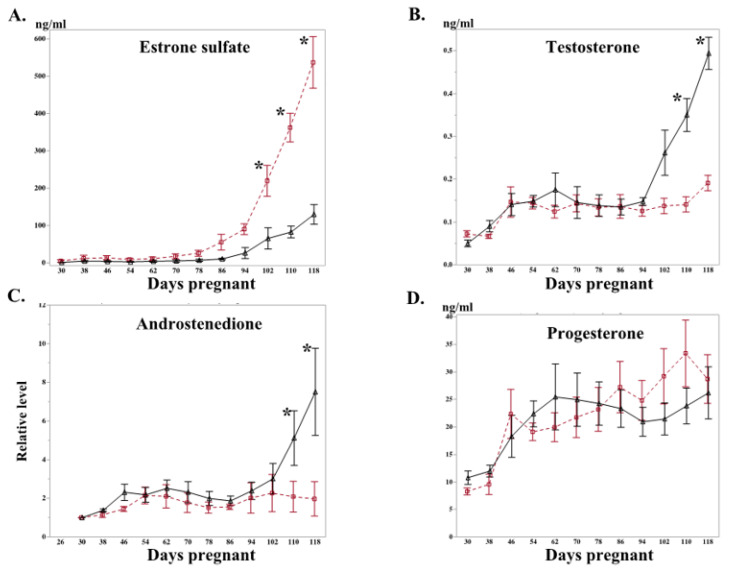

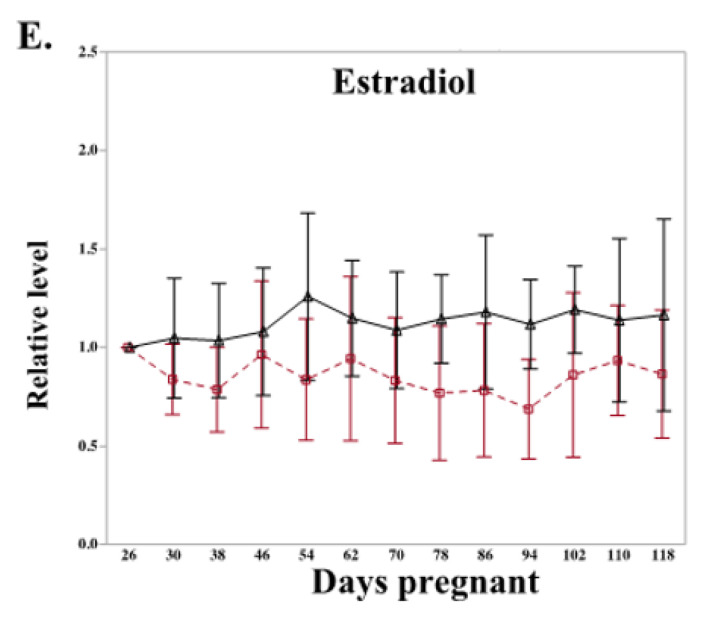
Sequential change in the level of reproductive hormones in maternal blood from letrozole (black triangle) and control (red square) groups. The values for androstenedione and estradiol were normalized to the first sample (D30 and D26 for androstenedione and estradiol, respectively) due to large individual variation. The day points that differ from the control are marked (* *p* < 0.05). (**A**) Estrone sulfate; (**B**) testosterone; (**C**) androstenedione; (**D**) progesterone; (**E**) estradiol.

Overall, maternal concentrations of estrone sulfate, testosterone, and progesterone increased with advancing gestational age (*p* < 0.05; Figure 2). Estrone sulfate concentrations were significantly lower in the letrozole-treated group than in the control mares, beginning at D102 (Figure 2A).

**Figure 3:**
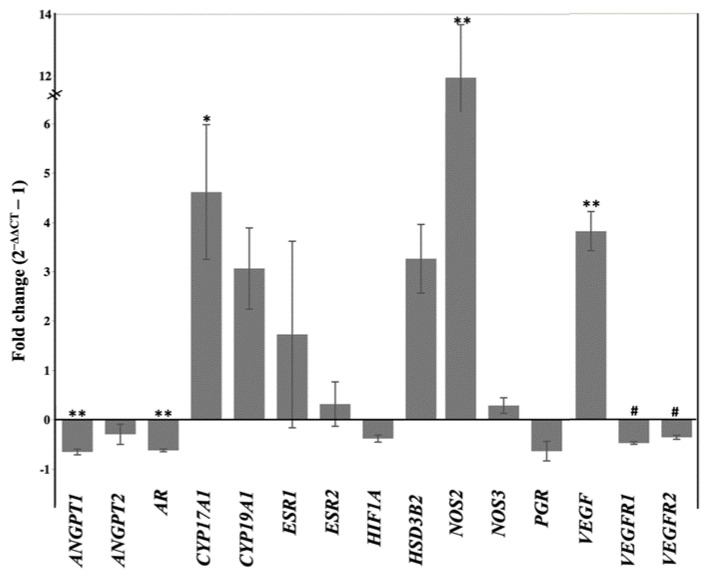
Gene expression (−∆CT values) in the chorioallantois for letrozole-treated and control groups. Data are presented as fold changes, setting the values from the controls as zero (* *p* < 0.05, ** *p* < 0.01, and ^#^ *p* < 0.1).

Conversely, peripheral testosterone and androstenedione concentrations were increased (*p* < 0.05) in letrozole-treated mares, beginning at D110, compared to the controls (Figure 2B and C, respectively). In line with this higher concentration of testosterone and androstenedione, expression of cytochrome P450 Family 17 *(CYP17A1;* converts 17-hydroxypregnenolone and 17-hydroxyprogesterone to dehydroepiandrosterone and androstenedione, respectively) was also increased in the chorioallantoises of letrozole-treated mares in comparison to those of the control mares (Figure 3). Moreover, the expression of androgen receptor *(AR)* in the chorioallantois was reduced in the letrozole-treated group (Figure 3).

The maternal serum concentrations of progesterone and estradiol were not affected by the treatment (Figure 2D,E). Similarly, the expressions of 3-beta hydroxysteroid dehydrogenase 2 (*HSD3B2*) and cytochrome P450 family 19 subfamily A member 1 (*CYP19A1*) were not affected by the treatment (Figure 3). The maternal serum concentration of eCG was also determined at D66 of gestation; no difference was observed between letrozole-treated and control mares (65.6 ± 6.8 vs. 73.5 ± 12.2 IU/mL, respectively). No difference was observed between the groups in the expression levels of steroid receptor signaling (estrogen receptors: *ESR1* and *ESR2,* progesterone receptor: *PGR*) in the chorioallantois (Figure 3).

### 2.3. Number of Vessels Tended to Decrease in Chorioallantois with Letrozole Treatment

**Figure 4:**
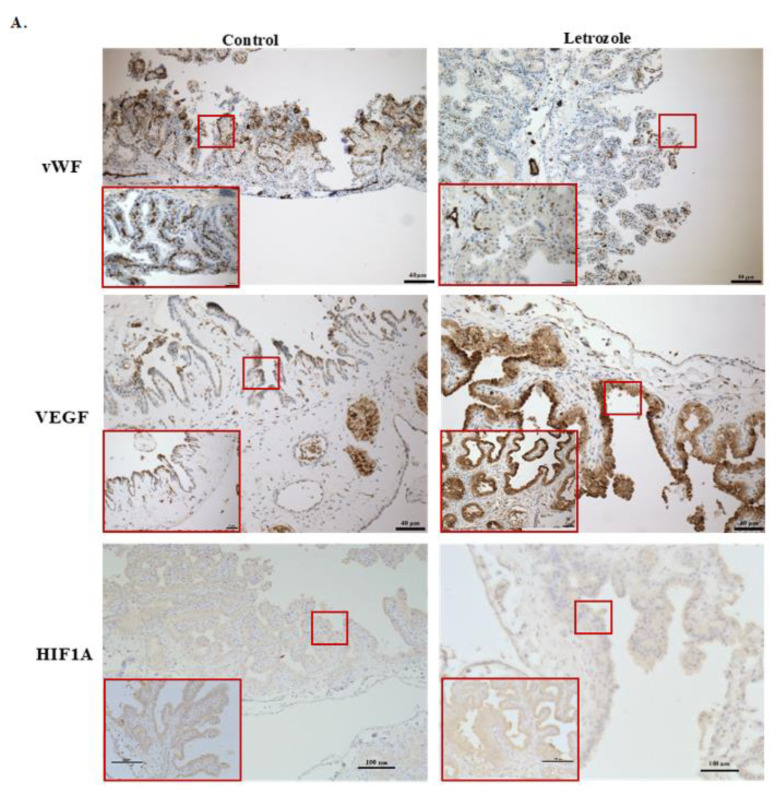

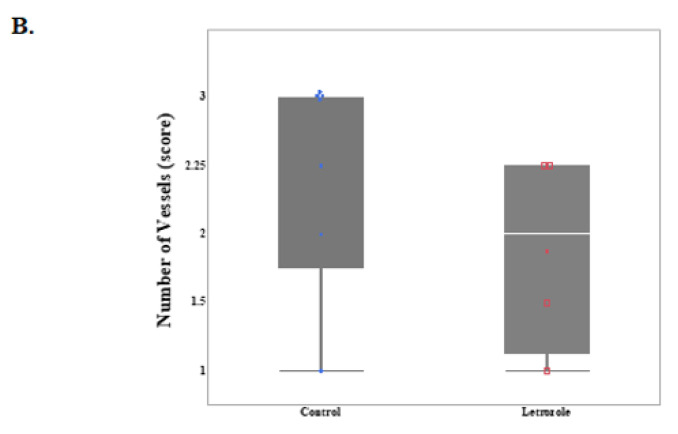
Immunohistochemical evaluation of letrozole-treated chorioallantoises (CA). (**A**) Immunohistochemical demonstration of capillary densities in chorioallantoic membranes of letrozole-treated and control mares using vWF. The capillary density tended to be greater in the control CAs than in the letrozole-treated CAs, *p* = 0.07; (**B**) A strong cytoplasmic signal for VEGF is noted within the chorionic epithelium of letrozole-treated group. No difference was observed in the abundance of HIF1A^+^ cells between the groups. (**B**) Red boxes (letrozole) and blue circles (control) represent values for the capillary density scores in the chorioallantoic samples.

Labeling with immunoreactive von Willebrand factor (vWF), a specific marker for endothelial cells, was compared between the treatment groups in the chorioallantois. The intensity of vWF was scored by two individuals blind to the treatment groups. The intensity of vWF tended to be higher in the control group’s chorioallantoises at D120 than in those of the letrozole group, indicating a reduced number of vessels (capillary intensity) in the letrozole-treated group (*p* = 0.07) (Figure 4).

We further evaluated the expression of a group of selected genes associated with angiogenesis, including *VEGF* and its receptors (*VEGFR1* and *VEGFR2*), *Angiopoietin 1* and *2* (*ANGPT1* and *ANGPT2*), and nitric oxide synthase (*NOS2 (iNOS)* and *NOS3 (eNOS)*) in the chorioallantois. *ANGPT1* showed a lower expression in the letrozole-treated chorioallantoises than in the controls (Figure 3). In contrast, *VEGF* and *NOS2* showed higher expressions in the letrozole-treated chorioallantoises than in the controls. Both VEGF receptors tended to have lower expression in letrozole-treated chorioallantoises than in the controls (*p* < 0.1; Figure 3). We further analyzed the correlation between the expression of these angiogenic genes and fetal weight and capillary intensity. There was a strong positive correlation between the expression of VEGFR receptors and capillary intensity (*r* = 0.76, *p* = 0.03) and between the capillary intensity and *VEGFR1* and *VEGFR2* (*r* = 0.78, *p* = 0.02). There was a negative correlation between the expression of *VEGF* and the weight of the fetuses (*r* = −0.68, *p* = 0.06).

Next, the immunoexpression of VEGF in the chorioallantois was compared between the groups via immunohistochemistry (IHC). Similar to gene expression, the intensity of immunoreactive VEGF within the trophoblast appeared greater in the letrozole-treated group than in the control group (Figure 4A).

It has been shown that low tissue vascularization and tissue hypoxia in the equine chorioallantois leads to overexpression of *VEGF* [12]. We hypothesized that the lower number of vessels in the letrozole-treated chorioallantoises might cause tissue hypoxia, leading to an increase in the expression of *VEGF.* Therefore, we evaluated the expression of hypoxia-inducible factor-1 alpha *(HIF1-α)* in the chorioallantois. There was no difference between the expression of *HIF1A* mRNA between the groups (Figure 3). Immunolocalization of HIF1A in the chorioallantois, like the gene expression of *HIF1A*, did not reveal any differences between the letrozole-treated group and the control group at the protein level, suggesting other pathways are involved in the overexpression of *VEGF* in the letrozole-treated chorioallantois (Figure 4).

No differences were observed in the expression levels of *ANGPT2* and *eNOS* in the chorioallantois between the groups (Figure 3).

### 2.4. 17β-Estradiol Increases the Angiogenic Activity of Equine Endothelial Cells

**Figure 5:**
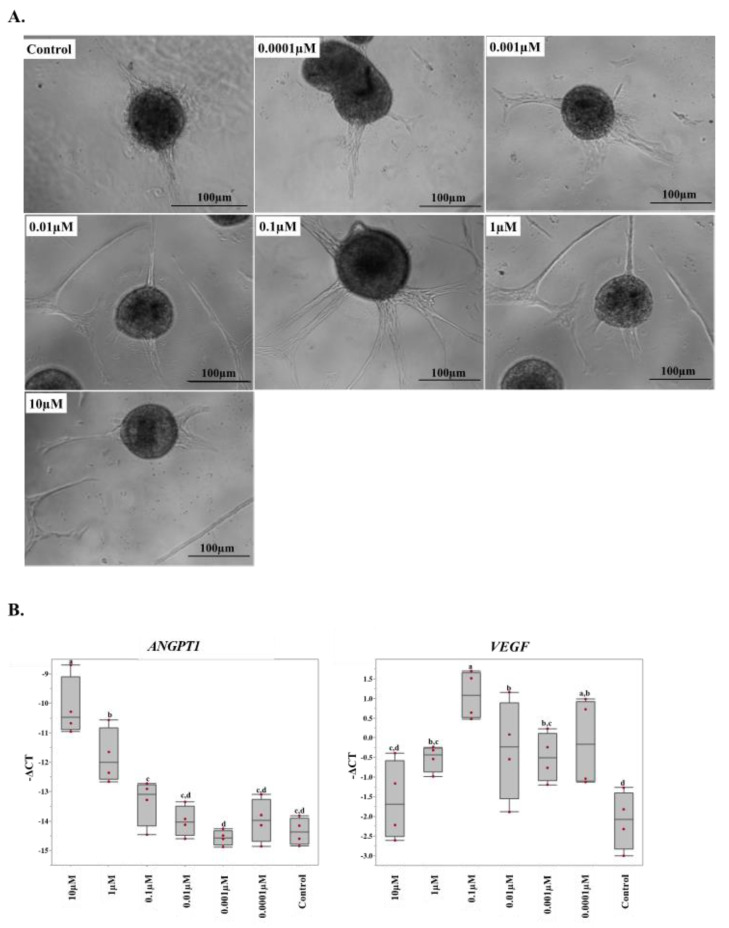
Angiogenic activity of equine endothelial cells after 17β-estradiol supplementation. (**A**) 17β-estradiol supplementation appears to induce higher rate of sprouting in the treatment groups than in the control cells. The 0.0001 µM, 0.001 µM, and control seem to have numerically fewer and shorter sprouts than the cells with the higher concentrations of 17β-estradiol supplementation. (**B**) The relative expression of angiogenic genes (−∆CT) measured by RT-qPCR in equine endothelial cells supplemented with different concentrations of 17β-estradiol. Different subscripts indicate statistical differences among the groups (*p* < 0.05).

To further understand the effect of estrogens on angiogenesis in the equine placenta, we evaluated the effect of different concentrations of 17β-estradiol (0.0001 µM to 1 µM) on equine endothelial cells. After estradiol treatment, spheroids were formed from the cells and their sprouting abilities were assessed as an indicator of angiogenesis. We found that 17β-estradiol induced endothelial cells to form sprouts and the degree of sprouting seemed to be greater with higher concentrations of estradiol (Figure 5A). Next, we analyzed the expression of the angiogenic genes in the estradiol-treated endothelial cells in vitro. The estradiol treatment had effects on the expression of ANGPT1; higher doses of 17β-estradiol caused a greater expression of ANGPT1 (Figure 5B). 17β-estradiol supplementation (0.0001 µM to 1 µM) also increased the expression of VEGF in the endothelial cells (Figure 5B). There was no difference in the expression of tested genes between the vehicles and the controls.

## 3. Discussion

Equine gestation is characterized by high concentrations of circulating estrogens in the mare. However, their physiological roles are largely unknown [1,2,3]. To investigate the role of estrogens in the equine pregnancy, we administered an aromatase inhibitor, letrozole, to the mares from D30 to D118 of gestation. The letrozole treatment decreased placental estrogen secretion, detected by a reduction in the maternal peripheral concentrations of estrone sulfate beginning at D102 of pregnancy. Testosterone and androstenedione, the precursors for estrogen synthesis, concurrently increased in the maternal blood, presumably as a result of aromatase inhibition. This increase in the androgen concentration may be responsible for the observed downregulation of *AR* gene expression in the CAs of the letrozole-treated group.

In this study, even though concentrations of estrone sulfate were significantly reduced in letrozole-treated mares beginning at D102, there was no decrease in peripheral concentrations of estradiol as a result of letrozole treatment. A similar treatment regime in late equine gestation (>8 months) reduced maternal estrogen concentrations by 90% [41]. Earlier studies of maternal concentrations of estrogens during early pregnancy demonstrated that maternal serum estradiol concentrations do not increase significantly until D108 to 150 of gestation [9,34]. Therefore, due to the low concentration, it was expected that there would be no detectable suppression in the maternal estradiol concentration prior to D120 in mares treated with letrozole.

In the current study, fetal size tended to be smaller in the letrozole-treated group than in the control group. This is in line with our previous study in which we demonstrated that neonates from letrozole-treated mares had lower birth weights than controls when the letrozole administration started at D240 of gestation until parturition [41]. However, this reduction in the size of the fetuses in both early and late gestation was not associated with uterine artery hemodynamics. As in the study during late-term gestation, we were unable to detect changes in the uterine artery diameter and blood flow between the treatment and control groups. The reduction of fetal size in the letrozole treatment group could be associated with an alteration in the placental function due to alteration in the placental microvasculature.

There are striking differences among eutherian mammals in the gross and microscopic structure of the placenta. However, regardless of these differences, the establishment of vascular networks in the placenta is a prerequisite for optimal placental function and normal fetal growth and development [9,10]. Several placental pathologies manifesting with abnormal fetal size are associated with abnormal vascular development in the placenta [11,12,13,14]. It has been suggested that angiogenic activities in reproductive tissues are highly regulated by steroid hormones [16,17]. In the present study, we found numerically fewer vessels in the chorioallantoises of the letrozole-treated mares than in those of the control mares. This alteration in the number of vessels, although not statistically significant, was accompanied by changes in the gene expression pattern of a group of angiogenic genes, including *ANGPT1* and *VEGF*.

VEGF is expressed throughout gestation in the luminal and glandular epithelia of the maternal endometrium and the trophoblast of the fetal chorioallantois [40]. In mice, disruption/inactivation of the genes encoding *VEGF* and its receptors causes embryonic lethality due to impaired/abnormal blood vessel formation [41,42,43,44]. When estradiol-17β was administered to ovariectomized ewes or baboons, endometrial *VEGF* mRNA was upregulated [45,46]. During pregnancy in baboons, *VEGF* mRNA was upregulated in parallel with increasing estradiol-17β levels; stimulation of estrogen secretion by androstenedione (A4) administration increased vessel density and *VEGF* mRNA in the primate placenta with associated increases in placental vascularization [47]. In contrast, estrogen suppression by letrozole in early pregnancy in baboons decreased cytotrophoblast *VEGF* mRNA expression. In healthy tissue, an increase in *VEGF* is accompanied by the upregulation of its receptor (*KDR*), leading to new vessel formation. However, in the present study, we found a high expression of *VEG*F along with a numerical downregulation of *VEGF’s* receptors. This could be associated with an impaired angiogenesis in the chorioallantoises of the treatment group, leading to a lower number of vessels in the placenta.

During tissue regeneration and development, local hypoxia leads to an increase in the expression of *VEGF* and its receptors [12]. Since VEGF was highly expressed in the trophoblastic layer of the treated group, we hypothesized that this could be associated with local hypoxia in this region. Therefore, we tested the expression of *HIF1A* as an indicator of tissue hypoxia. We were not able to identify any differences between the groups at the gene and protein level for this marker, suggesting that the lower number of vessels had not reached a critical point, leading to hypoxia. The other angiogenic gene that was differentially expressed in the chorioallantois of letrozole-treated mares was *ANGPT1*. *ANGPT1* contributes to blood vessel maturation and the stability of existing vessels [48,49]. The lower expression of *ANGPT1* in the treatment groups suggested an impaired vascular network due to a reduction in placental estrogens. In ovariectomized ewes, expression of *ANGPT1* was altered by estradiol-17β administration [50]. Moreover, administration of A4 to pregnant baboons also altered the *ANGPT1* mRNA level in cytotrophoblasts [47].

To test whether estrogens have direct effects on endothelial cells’ angiogenic activities, we treated equine endothelial cells with different concentrations of 17β-estradiol. As in our in vivo study, the expression of *VEGF* and *ANGPT1* was changed with the manipulation of 17β-estradiol. We found that supraphysiological concentrations of 17β-estradiol (1 and 10 ng/mL) increased the expression of *ANGPT1* in the endothelial cells. The expression of *VEGF* was also increased with supplementation of the media with 17β-estradiol. However, the expressions of VEGF receptors were not altered by 17β-estradiol supplementation. Similarly, the expression patterns of *ANGPT2*, *NOS2*, *NOS3*, *ESR1*, and *ESR2* were not affected by estradiol treatment. We further investigated the angiogenic ability of endothelial cells using a spheroid formation/sprouting assay. We found that 17β-estradiol supplementation could induce the endothelial cell spheroids to form sprouts, again suggesting its direct effect on vessel formation. An in-depth study to elucidate the molecular pathways involved in the role of estrogen in vessel formation is warranted.

## 4. Materials and Methods

### 4.1. Animals

Eleven clinically healthy mixed-breed mares, with a mean age of 10.4, were maintained at the Department of Veterinary Science’s Maine Chance Farm, University of Kentucky, Lexington, KY, USA. The mares were kept on pasture and were supplemented with grain and hay with free access to water and trace-mineralized salt. Mares were bred by artificial insemination, and ovulation (D0) was detected by transrectal ultrasonography. When the presence of a heartbeat was confirmed at D26, mares were divided into two groups with consideration of their age, parity, and body weight. Groups were randomly assigned to either letrozole-treated (*n* = 5; median age = 9, median parity = 0.5, and median body weight = 596) or control (*n* = 6; median age = 7, median parity = 0.5, and median body weight = 522) groups. Letrozole-treated mares received 500 mg letrozole (Aurum Pharmatech LLC., Howell, NJ, USA) orally every four days as previously established [41], and control mares received an equivalent mass of glucose orally every four days from D30 to 118 of gestation. Operators were blinded to the treatment identity for the duration of the study. All experimental procedures were approved by the Institutional Animal Care and Use Committee of the University of Kentucky (#2013-1067).

### 4.2. Ultrasonographic Analysis

For ultrasonographic evaluations, the operator was blinded to the treatment. Mares were evaluated every eight days from D26 to 114 of pregnancy. Mares were restrained in stocks without sedation, and fetal parameters (head length—distance from nose to occipital and uterine artery diameter) were assessed using transrectal, B-mode ultrasonography (SonoScape S8; SonoScape Medical Corp., Shenzhen, China) with a 7.5 MHz linear transducer. Uterine artery hemodynamics were determined using Doppler ultrasonography to measure the resistance index (RI). Briefly, the uterine arteries were localized by tracing the external iliac artery from the aorta to the divergence of the uterine artery and deep circumflex iliac arteries, approximately 10 cm from the aorta [43]. The uterine arteries of gravid and non-gravid uterine horns were analyzed separately.

### 4.3. Recovery of the Fetus and Placenta

At D120 of gestation, the fetus and placenta were collected transcervically from the control and treated mares. Briefly, mares were restrained in stocks, sedated, and the perineum and vulva were aseptically prepared. Cervical relaxation was achieved by intracervical administration of 2 mg prostaglandin E_2_ (PGE_2_) in triacetin and silicon dioxide gel [44]. Approximately 60 min after infusion of PGE_2_, the fetus and placental membranes were recovered transvaginally. After recovery, fetal weight, head length and crown–rump (C–R) length were determined. Samples of chorioallantois were stored in RNAlater (Thermo Fisher Scientific, Waltham, MA, USA) overnight at 4 °C, then moved to storage at −20 °C or fixed in 10% formalin solution, then transferred into methanol at 4 °C until further processing.

### 4.4. Endocrine Assays

Blood samples were collected by jugular venipuncture from mares, beginning at D26 and continuing every four days until D118. Blood was allowed to clot, and serum was separated and stored at −20 °C until endocrine analyses were performed. Serum concentrations of progesterone (P4), estrone sulfate (E1S), and testosterone (T) in maternal blood were determined in duplicates by previously validated enzyme immunoassays [41,48,49]. For progesterone, a previously validated competitive ELISA prepared in the laboratory [41,48] was used with a range of 0.02–20 ng/mL, using progesterone 3-*O*-carboxymethyloxime–horseradish peroxidase as the label and an antiserum raised in rabbits as a progesterone 11α-hemisuccinyl–bovine serum albumin immunogen. The limit of sensitivity of the progestin assay was 0.015 ng/mL. Intra- and inter-assay coefficients of variation (CV) for the progestin immunoassay were 7.5% and 13.1%, respectively. Serum estrone sulfate concentrations were determined with a competitive ELISA prepared in the laboratory, using an antibody which was produced in rabbits using oestrone-3-glucuronide conjugated to bovine serum albumin [41,49]. The standard curve ranged from 0.05 to 20 ng/mL, with a limit of detection of 0.049 ng/mL. Intra-assay and inter-assay CVs were 10.2% and 16.6%, respectively. For testosterone (T), a competitive ELISA was prepared and validated in our laboratory using a rabbit polyclonal anti-testosterone antibody (R156/7, Clinical Endocrinology Laboratory, UC Davis, Davis, CA, USA), testosterone: horseradish peroxidase (T:HRP, UC Davis), and testosterone for standards (Steraloids Inc., Newport, RI, USA). The standard curve ranged from 0.02 to 10 ng/mL with a limit of detection of 0.02 ng/mL. Intra- and inter-assay CVs were 11.9% and 18.1%, respectively [41]. Androstenedione (A4) concentrations in maternal blood were determined by ELISA (Abnova #KA1898; Walnut, CA, USA). Intra- and inter-assay CVs for A4 determinations were 9.3 and 10.2%, respectively. Concentrations of estradiol-17β (E2) in maternal blood were determined by a chemiluminescence immunoassay (IMMULITE 1000; Siemens, Munich, Germany). For the E2 automated assay, the standard curve ranged from 20–2000 pg/mL with an analytical sensitivity of 15 pg/mL. The intra- and inter-assay CVs were 1.2–1.4% and 2–3.15%, respectively. Concentrations of equine chorionic gonadotropin (eCG) were determined by immunoassay (BET Labs, Lexington, KY, USA).

### 4.5. Gene Expression Analysis

The expression of the genes related to angiogenesis and steroid hormone receptors (Table 1) in the chorioallantois at D120 were analyzed by RT-qPCR. Briefly, total RNA was extracted using TRIzol reagent (Thermo Fisher Scientific, Waltham, MA, USA) according to the manufacturer’s protocol. The concentration and quality of RNA were analyzed by spectrophotometry (NanoDrop 2000; Thermo Fisher Scientific). All extracted RNA had an absorbance value of more than 1.8 and 1.3 for 260/280 and 260/230, respectively. A two-microgram sample of RNA was treated with 1U of DNase (DNA-free Kit, Thermo Fisher Scientific, Waltham, MA, USA) and reverse transcribed into cDNA using 1.25 U/μL of MultiScribe reverse transcriptase (Thermo Fisher Scientific, Waltham, MA, USA) with 2.5 μM of random hexamers. The mixture was incubated at 25 °C for 10 min, followed by 48 °C for 30 min and 95 °C for 5 min. Targeted cDNAs were quantified by real-time PCR using a ViiA 7 system (Thermo Fisher Scientific, Waltham, MA, USA). RT-qPCR was performed using PowerUp SYBR Green Master Mix (Applied Biosystems, Waltham, MA, USA). The PCR reactions were performed in duplicate and were incubated at 95 °C for 10 min, followed by 45 cycles of 95 °C for 15 s and 60 °C for 1 min. The specificity of the PCR products was monitored via a melting curve and no fluorescence was detected in NTCs. The PCR efficiency was calculated using LinRegPCR (version 2012.0) (http://www.hartfaalcentrum.nl; accessed in 2 March 2019). The relative expression of each gene (∆CT) was calculated where ∆CT = (CT values of the gene of interest—geometric mean CT values of ACTB and GAPDH) [51,52].

### 4.6. Histological Analysis and Capillary Density Evaluation

For immunohistochemical staining, formalin-fixed paraffin-embedded (FFPE) tissue sections (5 microns) were mounted on positively charged Superfrost^®®^ Plus slides (Fisher Scientific, Pittsburgh, PA, USA). Immunostaining was performed using the BOND-MAX automated staining system and the BOND™ Polymer Refine Detection kit (Leica Biosystems, Buffalo Grove, IL, USA). Following automated deparaffinization, heat-induced epitope retrieval was performed using a ready-to-use citrate-based solution (pH 6.0; Leica Biosystems) at 100 °C for 20 min, and tissue sections were incubated with a rabbit anti-von Willebrand factor (vWF) antibody (Dako, Carpinteria, CA, USA; 1:1000 dilution in IHC/ISH SuperBlocking buffer; Leica Biosystems) for 1 h at room temperature, followed by a polymer-labeled goat anti-rabbit IgG conjugated with horseradish peroxidase for 8 min at room temperature. 3,3′ diaminobenzidine was used as the substrate and incubated for 10 min. Finally, counterstaining was performed with hematoxylin (5 min) and slides were mounted using a permanent mounting medium (Micromount^®®^, Leica Biosystems, Buffalo Grove, IL, USA). Moreover, immunohistochemistry was performed to detect the expression and localization of a vascular endothelial growth factor (VEGF) and hypoxia-inducible factor-1 alpha (HIF1A) in the chorioallantois as described above using mouse anti-human VEGF (1:50, #MA5-13182; Thermo Fisher Scientific, Waltham, MA, USA) and mouse anti-human HIF1A (1:200; #MA1-16504; Thermo Fisher Scientific, Waltham, MA, USA). Normal mouse and rabbit IgG were used as isotype controls.

The number of vessels (capillaries and small venules distinctively identified as von Willebrand factor-positive and irrespective of complete or incomplete lumen due to plane of section or presence of erythrocytes within the lumen) was determined using the vascular hotspot method (Weidner’s method), in which the five areas with the highest density of vessels (hotspots) are selected, and vessel counting is performed at high magnification [12,45,46,53]. The numbers of vessels were evaluated and scored by two different investigators blinded to the experiment, using a Zeiss microscope equipped with a MicroPublisher 5.0 RTV camera (Q-Imaging, Burnaby, BC, Canada). For quantification purposes, sections were scored as follows: 0, no staining; 1, weak or minimal staining; 2, mild staining; 3, intense or abundant staining.

### 4.7. Evaluating the Effect of 17β-Estradiol on Equine Endothelial Cells

Endothelial cells (derived from neonatal foal pulmonary artery endothelial cells; University of California, Davis, CAEEC [47]) were cultured in Dulbecco’s modified Eagle’s Medium (DMEM, Thermo Fisher Scientific, Waltham, MA, USA) with 10% fetal bovine serum (FBS, Hyclone Laboratories, Inc., Logan, UT, USA), 100 U/mL penicillin–streptomycin, 1 mM sodium pyruvate, 0.1 mM non-essential amino acids, and 200 mM l-glutamine (Thermo Fisher Scientific, Waltham, MA, USA) in a humidified incubator at 37 °C with 5% CO_2_. Cells were cultured in 6-well plates (0.3 × 10^6^ seeding density), and wells were treated with different concentrations of 17β-estradiol (Sigma Aldrich, St. Louis, MO, USA), vehicle or no treatment for three days. 17β-estradiol was initially dissolved in 100% ethanol (100 mM stock) and then subsequently diluted to 10 mM in 50% ethanol. The 10 mM stock was then used to make a serial dilution (10 µM, 1 µM, 0.1 µM, 0.01 µM, 0.001 µM, and 0.0001 µM) using distilled water.

After the third day, the cultured endothelial cells were trypsinized and re-suspended at a density of 10^4^ cells per 10 mL in the aforementioned culture medium that was supplemented with 20% Methocell (Sigma Aldrich, St. Louis, MO, USA) to increase the viscosity of the medium [54]. Subsequently, the cell suspensions were adhered as hanging drops of 50 µL to the lids of square Petri dishes. This allowed the cells to cluster at the tips of the drops and to form spheroids. After 24 h, each drop contained one spheroid, consisting of approximately 500 cells. These spheroids were collected and suspended at a concentration of 50 spheroids per ml in the aforementioned medium with 30% Methocell and 2.5 mg/mL rat tail collagen type I (Sigma Aldrich, St. Louis, MO, USA). After 48 h, all spheroids were visualized with an inverted microscope. The experiment was repeated three times, and each estradiol concentration was run in eight replicates.

### 4.8. Statistics

Data were assessed for normality using a Kolmogorov–Smirnov test and for equal variance using Bartlett’s test. Non-normally distributed data were transformed using normal quantile transformation [50]. The statistical analysis was performed on the normalized data. Hormone concentrations and ultrasonographic data were analyzed using two-way repeated measures ANOVA with post hoc comparisons via the Tukey–Kramer method. Data for serum concentrations of E2 and A4 showed large variation between animals, and these data were normalized to pretreatment values for comparisons of relative changes during treatment. Fetal parameters and gene expressions were compared using the non-parametric, Mann–Whitney U test. Capillary densities in histological sections were compared using a t-test. RT-qPCR data (−∆CT values) were compared between the letrozole-treated and control groups using a t-test. Gene expressions (−∆CT values) were also compared among the different estradiol concentrations using ANOVA with post hoc comparisons via the Tukey–Kramer method.

## 5. Conclusions

In conclusion, we demonstrated possible effects of estrogens by substrate competitive suppression using letrozole during the first trimester of equine pregnancy. Fetal weight in the estrogen-suppressed group tended to be lower, suggesting an impaired placental vasculature network. This was accompanied with alterations in the expression of *VEGF* and *ANGPT1* in the chorioallantoises of the letrozole-treated group. The altered gene expression was also manifested in a tendency to have a lower vessel density in the estrogen-suppressed chorioallantois. We were also able to demonstrate the effect of estrogens on the gene expression of *ANGPT1* and *VEGF*, along with changes in the angiogenic activity of equine endothelial cells, in our in vitro study. Overall, we suggest that estrogen-dependent vasculogenesis in the equine chorioallantois affects equine fetal development.

## Figures and Tables

**Table 1:** The list of primers used in this study.

Transcript	NCBI ID		Sequence of Primer (5′-3′)	Product Size (bp)
*ACTB*	100033878	*F:*	CGACATCCGTAAGGACCTGT	100
		*R:*	CAGGGCTGTGATCTCCTTCT	
*ANGPT1*	100056482	*F:*	GGACAGCAGGAAAACAGAGC	93
		*R:*	GGGCACATTTGCACATACAG	
*ANGPT2*	100051890	*F:*	GACGCAGACAACGACAAATG	76
		*R:*	GACCACATGCATCAAACCAC	
*AR*	100033980	*F:*	AGCTGCCATCCACTCTGTCT	61
		*R:*	TGATAAACTGCTGCCTCGTC	
*CYP17A1*	100034232	*F:*	GCATGCTGGACTTACTGATCC	60
		*R:*	CTGGGCCAGTGTTGTTATTG	
*CYP19A1*	100009712	*F:*	CCACATCATGAAACACGATCA	60
		*R:*	TACTGCAACCCAAATGTGCT	
*ESR1*	791249	*F:*	TCCATGGAGCACCCAGGAAAGC	125
		*R:*	CGGAGCCGAGATGACGTAGCC	
*ESR2*	100033964	*F:*	TCCTGAATGCTGTGACCGAC	116
		*R:*	GTGCCTGACGTGAGAAAGGA	
*GAPDH*	100033897	*F:*	AGAAGGAGAAAGGCCCTCAG	88
		*R:*	GGAAACTGTGGAGGTCAGGA	
*HIF1A*	100061166	*F:*	CACCAGAGCCTAACAGTCCC	141
		*R:*	AGTCCGTGTCCTGAGTGGAA	
*HSD3B2*	100034078	*F:*	AGCAAATACCATGAGCACGA	62
		*R:*	TAACGTGGGCATCTTGTGAA	
*NOS2*	791246	*F:*	GCCAAGGTCTGAGCTACCTG	200
		*R:*	GAGTGCCTGGCTGAGTGAG	
*NOS3*	100063339	*F:*	GAAGCACCTGGAGAATGAGC	150
		*R:*	TCTGGCTGGTAGCGGAAG	
*PGR*	100033883	*F:*	GTGAGAAGGGAAGTGGAAC	226
		*R:*	GGAGGGCAGGAGAAGGTAGT	
*VEGF*	100033839	*F:*	CTACCTCCACCATGCCAAGT	88
		*R:*	GACGTCCATGAACTTCACCA	
*VEGFR1*	100033957	*F:*	GGCACAAAGACCCAAAAGAA	88
		*R:*	CCGTCCTGTTGTACATTTGC	
*VEGFR2*	100033959	*F:*	AGATGCTGTAACCCGGAGTG	79
		*R:*	CGTGCTGTTCTTCTTGGTCA	

## Data Availability

Not applicable.
